# Effects of *Citri Reticulatae Pericarpium* powder on the baking quality and volatile flavor compounds of toast bread

**DOI:** 10.3389/fnut.2026.1964585

**Published:** 2026-10-06

**Authors:** Yongfeng Jiang, Han Yang, Yuanyuan Hu, Yaoxu Lin, Enrui Hu, Qingjie Shi, Jinxiang Hu, Hao Zhang, Yuwen Yi

**Affiliations:** 1 School of Food Engineering, Guangdong Teachers College of Foreign Languages and Arts, Guangzhou, China; 2 College of Culinary and Food Science Engineering, Sichuan Tourism University, Chengdu, China; 3 Sichuan Digital Economy Industry Development Research Institute, Chengdu, China; 4 Cuisine Science Key Laboratory of Sichuan Province, Sichuan Tourism University, Chengdu, China

**Keywords:** *Citri Reticulatae Pericarpium* powder, electronic nose, odor activity value, toast bread, volatile flavor compounds

## Abstract

**Background:**

*Citri Reticulatae Pericarpium* (CRP) is rich in terpene volatiles, particularly D-limonene, and has a distinctive citrus-like aroma. However, its effects as a natural flavoring ingredient on the baking quality and volatile aroma profile of toast bread remain insufficiently characterized. This study evaluated the effects of CRP powder addition on baking quality and aroma characteristics and screened potential odor-active compounds associated with different addition levels.

**Methods:**

CRP powder partially replaced high-gluten wheat flour at levels of 0, 1.0, 2.0, and 3.0%. Baking quality attributes were evaluated by measuring specific volume, loaf height, baking loss, color, and texture. Aroma characteristics were assessed by sensory analysis and an electronic nose, while volatile compounds were analyzed by gas chromatography–mass spectrometry (GC–MS). Odor activity values (OAVs) and partial least squares discriminant analysis (PLS-DA) were used to characterize potential odor-active compounds and differences among treatments.

**Results:**

Increasing CRP powder reduced specific volume from 2.98 to 2.54 cm^3^/g and increased hardness from 7.41 to 9.37 N and chewiness from 47.18 to 57.80 mJ. Crumb lightness decreased, whereas yellowness increased. Sensory analysis showed a progressive enhancement of characteristic CRP aroma, with the 2.0% group exhibiting relatively favorable aroma harmony, purity, and fullness. Electronic-nose PCA clearly separated the control from CRP-supplemented samples. GC–MS detected 33 volatile compounds. Terpenes became the dominant volatile class after CRP addition, reaching 3,024.16, 6,745.46, and 8,426.43 μg/kg in the 1.0, 2.0, and 3.0% groups, respectively. D-limonene was the predominant terpene, with OAVs of 82.59, 184.00, and 222.72, suggesting that it was a major potential odor-active contributor to the characteristic CRP aroma. PLS-DA based on OAV data effectively discriminated the four groups.

**Conclusion:**

CRP powder substantially reshaped the aroma profile of toast bread but adversely affected loaf expansion and texture at higher addition levels. Overall, 2.0% CRP powder provided a favorable compromise between enhancement of the characteristic CRP aroma and the accompanying changes in baking quality, supporting its potential application as a natural flavoring ingredient in bakery products.

## 1 Introduction

Toast bread is a typical fermented bakery product prepared mainly from wheat flour, yeast, water, sugar, and fat through mixing, fermentation, molding, proofing, and baking. Aroma is an important sensory attribute influencing consumers’ perception of toast bread quality, and its formation occurs throughout both fermentation and baking. During fermentation, yeast metabolism generates alcohols, esters, aldehydes, and their precursors (1, 2). During baking, Maillard reactions, lipid oxidation, and thermal degradation further promote the formation of various volatile compounds, jointly producing the characteristic fermentation and baked aromas of bread (2). Previous studies have shown that yeast dosage, fermentation temperature, and yeast strain can significantly affect the formation of volatile compounds and the overall aroma profile of wheat bread (3–5). In addition, modulation of the sourdough microbiota and fermentation conditions can further alter bread aroma characteristics (6, 7).

Current approaches to improving bread aroma have focused largely on fermentation regulation, such as sourdough and mixed-culture fermentation (6, 7), whereas the direct use of natural plant-derived ingredients to modulate bread aroma has received comparatively less attention. Orange peel powder has been reported to alter the rheological properties of wheat dough and bread quality (8). Valorization of citrus peel can improve the bioaccessibility of phenolic compounds and antioxidant potential in bread (9), while the incorporation of fruit-derived dietary fiber can also alter the volatile compound profile of bread (10). However, most existing studies have focused on the effects of citrus by-products on dough processing properties, nutritional quality, and antioxidant activity, and their effects on the volatile flavor composition and characteristic aroma formation of bread remain insufficiently investigated.

*Citri Reticulatae Pericarpium* (CRP) is a characteristic food ingredient produced by drying and aging mature citrus peel. Owing to its distinctive aroma and chemical composition, CRP has attracted considerable attention in food and related product development (11). The volatile fraction of CRP is dominated by monoterpenes and sesquiterpenes, with D-limonene generally being the predominant volatile constituent; γ-terpinene, β-pinene, and p-cymene are also important components (12, 13). In addition, geographical origin, variety, and aging time can influence the chemical composition and flavor characteristics of CRP (14, 15), indicating that CRP possesses a complex volatile basis as a natural flavoring ingredient.

Studies directly incorporating CRP into bakery products to modulate volatile flavor remain limited. In particular, little information is available on the relationship between CRP powder addition level and changes in toast bread aroma across multiple levels, including sensory perception, overall odor profile, and potential odor-active compounds. Therefore, in this study, CRP powder was used to partially replace wheat flour at different levels to prepare toast bread. Sensory evaluation, electronic-nose analysis, gas chromatography–mass spectrometry (GC–MS), odor activity value (OAV) analysis, and partial least squares discriminant analysis (PLS-DA) were combined to systematically evaluate the effects of CRP powder addition on the baking quality attributes, volatile composition, and aroma characteristics of toast bread, with the aim of providing a theoretical basis for the application of CRP as a natural flavoring ingredient in bakery products.

## 2 Materials and methods

### 2.1 Materials and reagents

#### 2.1.1 Materials

Lanyue bread flour, Yihai Kerry Arawana Holdings Co., Ltd.; Xinhui Meijiang *Citri Reticulatae Pericarpium* (2025), purchased from a CRP retailer in Xinhui District, Jiangmen, China; Jiuda low-sodium salt, Sichuan Jiuda Salt Co., Ltd.; Angel semi-dry yeast and Angel bread improver, Angel Yeast Co., Ltd.; sucrose, Guangdong Zhongqing Sugar Industry Group; Nestlé skim milk powder, Shuangcheng Nestlé Co., Ltd.; Président unsalted butter, Lactalis Group, France.

#### 2.1.2 Reagents

2-Methyl-3-heptanone (CAS No. 13019–20-0), Sigma-Aldrich Trading (Shanghai) Co., Ltd.; C7-C40 n-alkane mixed standard (chromatographic grade), Sigma, USA.

### 2.2 Instruments and equipment

TMS-Pro texture analyzer, FTC, USA; NR200 colorimeter, Shenzhen 3nh Technology Co., Ltd., China; FOX 4000 electronic nose, Alpha MOS, France; HST40 trap headspace sampler coupled with a Clarus 680 gas chromatograph and SQ8T mass spectrometer equipped with an Elite-5MS column (30 m × 0.25 mm × 0.25 μm), PerkinElmer, USA. Other routine laboratory apparatus and glassware were also used.

### 2.3 Experimental methods

#### 2.3.1 Preparation of toast bread samples

##### 2.3.1.1 Preparation of CRP powder

The white pith was removed from the CRP, after which the peel was dried in an oven at 60 °C to constant weight (16), ground, and passed through a 100-mesh sieve. The resulting CRP powder was sealed and stored until use.

##### 2.3.1.2 Preparation of toast bread

Toast bread was prepared using the rapid straight-dough method for pan bread described in reference (17), with minor modifications. Preliminary trials were conducted with CRP powder replacement levels of 0, 0.5, 1.0, 1.5, 2.0, 2.5, and 3.0%. Based on dough-forming characteristics, specific volume, loaf height after baking, and aroma performance, 0, 1.0, 2.0, and 3.0% were selected for the subsequent experiments and designated as groups A, B, C, and D, respectively. The formulation, expressed as baker’s percentages, consisted of high-gluten wheat flour and CRP powder (100% in total), semi-dry yeast (1.3%), bread improver (0.3%), salt (2%), sucrose (8%), skim milk powder (4%), butter (8%), and water (60%). CRP powder replaced high-gluten wheat flour at the designated levels while the total proportion of flour plus CRP powder was maintained at 100%. All other ingredient proportions and processing conditions were kept constant, and 510 g of dough was placed in each toast mold. Bread flour, CRP powder, and the other dry ingredients were mixed thoroughly, followed by addition of the yeast suspension and water to form a dough. Butter was then incorporated, and mixing continued until full dough development. The dough temperature after mixing was maintained at approximately 30 °C. The dough was rested for 20 min, divided and pre-shaped, subjected to an intermediate proof for 10 min, then finally shaped and placed in toast molds. Final proofing was performed at 35–38 °C and 85% relative humidity until the dough reached approximately 80% of the mold height. Baking was then carried out at 190 °C top heat and 210 °C bottom heat for 35–40 min. After baking, the loaves were immediately removed from the molds and cooled at room temperature for 1 h. They were then sliced to a thickness of 25 mm, and the subsequent sensory, color, texture, electronic-nose, and GC–MS analyses were initiated immediately without additional storage. Three replicate samples were prepared for each group.

### 2.4 Analytical methods

#### 2.4.1 Sensory evaluation

A structured scoring method was used to evaluate the aroma characteristics of the samples. The sensory panel consisted of 11 assessors with prior experience in food sensory evaluation. Before formal evaluation, the assessors received brief training to familiarize them with the definitions of the sensory attributes and the scoring procedure. All evaluations were conducted in a quiet room with uniform lighting and no obvious extraneous odors at 20–25 °C. Samples were labeled with random three-digit codes, and the assessors completed the evaluations independently without discussion. Each sample was evaluated three times. The evaluation criteria are presented in Table 1. All attributes were scored on a 1–9 scale. For characteristic CRP aroma intensity and baked aroma intensity, 1 represented very weak and 9 represented very strong; for aroma harmony, purity, and fullness, 1 represented the lowest level and 9 represented the highest level. In accordance with institutional regulations, the relevant ethics committee granted a waiver for formal ethical review, and written informed consent was obtained from all participants.

**Table 1:** Sensory evaluation criteria for toast bread.

Sensory attribute	Description
Characteristic CRP aroma intensity	Intensity of the typical CRP aroma in the sample; the aroma should be identifiable and natural rather than harsh or obtrusive.
Baked aroma intensity	Intensity of the pleasant baked, wheaty, and fermentation-derived aromas characteristic of toast bread.
Aroma harmony	Degree of integration between CRP aroma and the intrinsic baked and fermented aromas of toast bread, including naturalness, harmony, and overall coherence.
Aroma purity	Cleanliness and purity of the sample aroma, including the absence of unpleasant medicinal or other off-odors.
Aroma fullness	Overall richness, fullness, and roundness of the aroma, as opposed to thinness or hollowness.

All sensory attributes were scored on a 1–9 scale, where 1 indicated the lowest level of the attribute and 9 indicated the highest. Characteristic CRP aroma and baked aroma were rated from very weak to very strong, whereas aroma harmony, purity, and fullness were rated from low to high. The sensory panel consisted of 11 trained assessors, and samples were independently evaluated using random three-digit codes.

#### 2.4.2 Determination of specific volume, texture, color, baking loss, loaf weight, and loaf height

Specific volume, texture, and color were measured according to the methods of Alkandari et al. (18). Baking loss was determined according to Kinner et al. (19). Loaf height was measured at the highest point in the center of each toast loaf using a ruler.

#### 2.4.3 Electronic-nose analysis

##### 2.4.3.1 Sample preparation

A central slice of each toast loaf was collected, avoiding the first and last slices. The crumb was ground and thoroughly mixed, and 1.00 g was rapidly transferred into a 10 mL headspace vial, which was immediately sealed. Sampling location and pretreatment were kept consistent among all groups.

##### 2.4.3.2 Analytical conditions

The headspace incubation temperature was 70 °C, the equilibration time was 300 s, and the injection volume was 1,000 μL. Electronic-nose parameters were as follows: manual injection rate, 1,000 μL/s; analysis time, 120 s; and sensor cleaning time, 180 s. Each group was measured repeatedly, and the stable sensor signals recorded at 120 s with a relative standard deviation (RSD) ≤ 5% were used for subsequent analysis.

#### 2.4.4 GC–MS analysis

##### 2.4.4.1 Sample preparation

A central slice of each toast loaf was collected, avoiding the first and last slices. The sample was ground and thoroughly mixed, and 3.00 g was placed in a 20 mL headspace vial. Subsequently, 20 μL of 2-methyl-3-heptanone internal standard solution (4.075 mg/mL) was added, and the vial was immediately sealed for subsequent GC–MS analysis.

##### 2.4.4.2 Extraction and GC–MS conditions

The extraction temperature was 70 °C and the extraction time was 30 min. The needle temperature was 75 °C and the transfer-line temperature was 80 °C. Dry purge was performed for 2 min, desorption for 0.1 min, and pressurization/depressurization for 2 min. The trap was held for 4 min, and four trap cycles were performed. The injector temperature was 250 °C. Helium was used as the carrier gas at a constant flow rate of 0.8 mL/min in splitless mode. An Elite-5MS column (30 m × 0.25 mm × 0.25 μm) was used. The initial oven temperature was 60 °C and held for 2 min, then increased to 200 °C at 10 °C/min with no holding time, followed by an increase to 300 °C at 20 °C/min and a final hold of 3 min (20).

Electron ionization (EI) was performed at 70 eV. The ion-source temperature was 230 °C, and the mass scan range was m/z 45–550.

##### 2.4.4.3 Qualitative and semi-quantitative analysis

A C7-C40 n-alkane mixed standard was analyzed by GC–MS under the chromatographic conditions described in Section 2.4.4.2 to obtain the retention times of the n-alkanes. Retention indices (RIs) were calculated according to the method described in reference (21).

For qualitative GC–MS analysis, compounds were searched against the NIST 2011 mass spectral library, and identifications with both forward and reverse match scores ≥800 were retained. For compounds containing more than seven carbon atoms, RI confirmation was additionally applied when reliable reference RI data were available. A compound was considered reliably identified when the deviation between the experimentally calculated RI and the literature RI was within ±50. Compounds lacking reliable reference RI data were retained as tentative identifications based on mass spectral matching.

Volatile compounds were semi-quantified using 2-methyl-3-heptanone as a single internal standard, with minor modifications to the procedure of Liu et al. (22). In the absence of compound-specific response factors, a relative response factor of 1.0 was assumed for all analytes. Therefore, the resulting concentrations were regarded as semi-quantitative estimates and were used primarily for comparison of volatile levels among treatment groups rather than as absolute concentrations determined from compound-specific calibration curves.

##### 2.4.4.4 Odor activity value analysis

The odor activity value (OAV) of each volatile compound was calculated according to Eq. (1):

OAVi=Ci/OTi (1)

where C_i_ is the semi-quantitative concentration of volatile compound i (μg/kg), and OT_i_ is the corresponding odor threshold (μg/kg). Compounds with OAV_i_ > 1 were considered potential odor-active compounds that may contribute to the overall aroma of the samples (22). Because C_i_ was derived from single-internal-standard semi-quantification rather than compound-specific calibration, the calculated OAVs were used primarily for preliminary screening of potential odor-active compounds and for relative comparison among treatment groups, rather than for precise quantification of the absolute aroma contribution of individual compounds.

### 2.5 Correlation analysis between electronic-nose responses and volatile compounds

To further investigate the relationship between electronic-nose responses and major volatile compounds, Pearson correlation analysis was performed using 12 matched independent samples (A1–A3, B1–B3, C1–C3, and D1–D3), based on the original stable responses of the 18 electronic-nose sensors and the semi-quantitative data of the selected volatile compounds. A two-step screening strategy was applied to select volatile compounds. First, potential odor-active compounds with OAV > 1 in at least one treatment group were included. Considering that electronic-nose sensor responses are also influenced by volatile abundance and physicochemical properties, major differential volatile compounds that were detected in at least two treatment groups, had a semi-quantitative concentration ≥100 μg/kg in at least one group, and differed significantly among groups (*p* < 0.05) were additionally included. A total of 15 volatile compounds were selected, and non-detected compounds were assigned a value of 0.

### 2.6 Statistical analysis

Raw data were organized and calculated using Excel 2019. One-way analysis of variance (ANOVA) and significance testing were performed using SPSS 26.0, with Duncan’s multiple-range test used for pairwise comparisons among groups at *p* < 0.05. Origin 2021 was used to generate sensory and electronic-nose plots. Raw electronic-nose sensor data were normalized before heat-map visualization, and the same original dataset was subjected to dimensionality reduction for principal component analysis (PCA). Partial least squares discriminant analysis (PLS-DA) and variable importance in projection (VIP) screening were performed using SIMCA 14.1 to characterize differences in OAV profiles and screen differential OAV variables among treatment groups. For PLS-DA, OAVs of replicate samples from each treatment group were used as the variable matrix. Non-detected compounds were assigned a value of 0, while detected compounds with OAV < 1 were included using their actual calculated values. Model stability was evaluated using 200 permutation tests.

## 3 Results and discussion

### 3.1 Sensory evaluation

As shown in Figure 1, the CRP powder addition level had a marked effect on the aroma quality of toast bread. The intensity of the characteristic CRP aroma increased significantly with increasing CRP powder addition, with lower scores in the 0 and 1% groups and significantly higher scores in the 2 and 3% groups (*p* < 0.05), indicating that CRP powder effectively imparted its characteristic aroma to the toast bread. Baked aroma intensity varied little among groups and remained at a relatively high level, suggesting that moderate CRP powder addition did not markedly weaken the inherent baked aroma of toast bread. In terms of aroma harmony and purity, the 2% group performed relatively well, suggesting more natural integration of CRP aroma with the intrinsic baked and fermented aroma of toast bread at this addition level. Both aroma harmony and purity were lower in the 3% group than in the 2% group, suggesting that excessive CRP powder addition reduced the integration and cleanliness of the overall aroma profile. Overall, the 2% group achieved a favorable balance among characteristic CRP aroma, harmony, purity, and fullness and was therefore considered a relatively suitable addition level.

**Figure 1:**
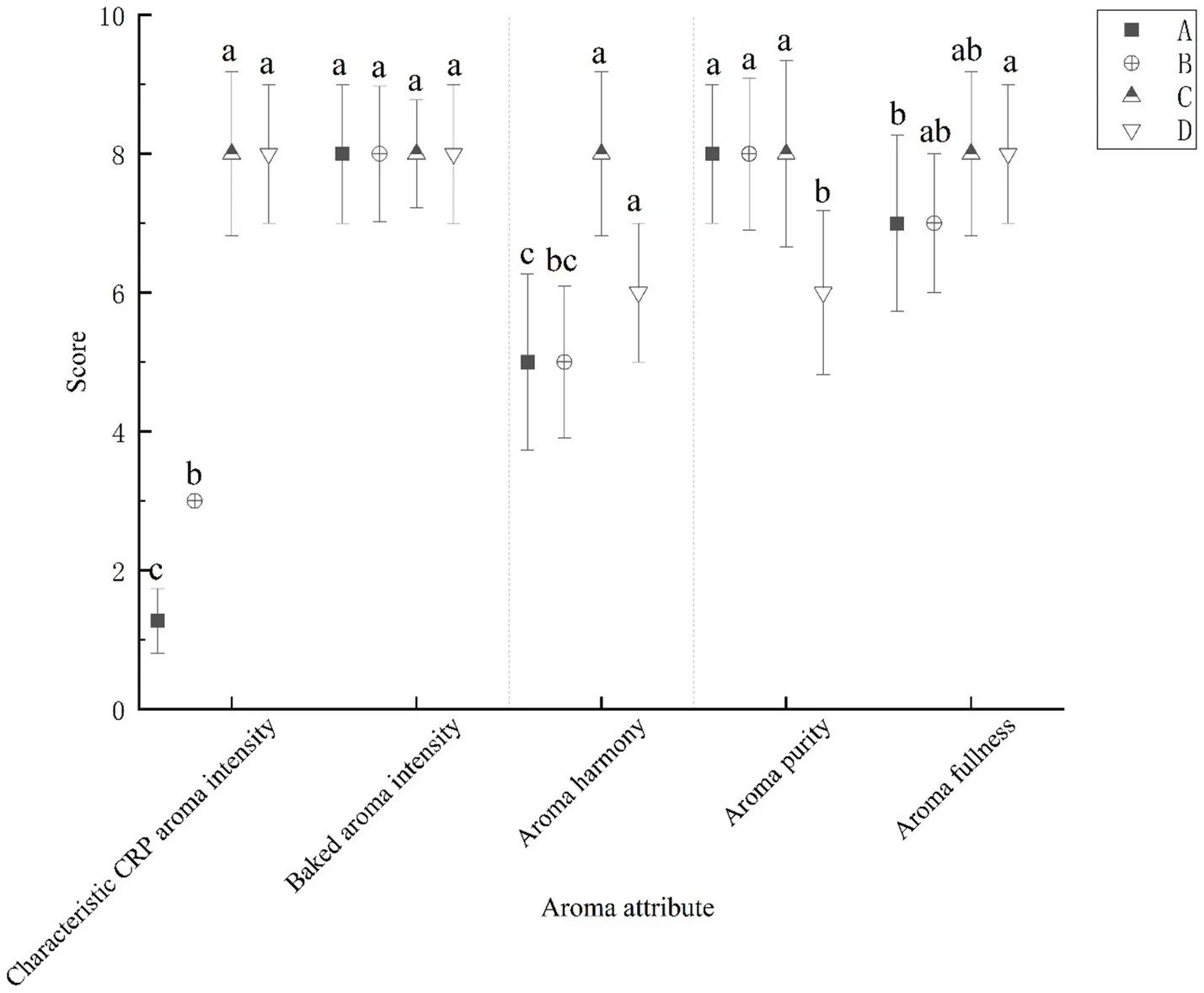
Scatter plot of sensory evaluation. A, B, C, and D represent toast bread samples containing 0, 1.0, 2.0, and 3.0% CRP powder, respectively. Points represent the mean sensory scores of each treatment group, and error bars represent standard deviations. Within the same sensory attribute, different lowercase letters indicate significant differences among treatment groups (*p* < 0.05), whereas values sharing at least one lowercase letter are not significantly different (*p* ≥ 0.05).

### 3.2 Specific volume, loaf height, loaf weight, and baking loss

As shown in Table 2, CRP powder addition markedly affected the baking quality attributes of toast bread. Specific volume and loaf height generally decreased with increasing CRP powder addition. No significant differences were observed between the 0 and 1% groups, whereas both indices decreased significantly in the 2 and 3% groups, indicating that a low level of CRP powder had little effect on loaf expansion but higher addition levels were unfavorable for volume development. The similar trends in specific volume and loaf height suggest that CRP powder may have affected dough leavening and gas-retention capacity. This effect is likely related to dilution of gluten proteins when CRP powder partially replaces wheat flour and to changes in water distribution and gluten network formation caused by dietary fiber, pectin, and other polysaccharides in CRP powder. Previous studies have shown that orange peel powder can significantly affect dough rheology and fermentation characteristics, mainly through competitive water absorption, gluten dilution, and reduced gas-retention capacity caused by fiber, pectin, and polyphenols (8). From the perspective of dietary fiber-gluten protein interactions, exogenous fiber can compete with gluten proteins for water, form hydrogen bonds and other non-covalent interactions, and alter protein secondary structure and disulfide-bond conformation, thereby affecting gluten protein aggregation and formation of a continuous gluten network (23). The effect of citrus fiber on bread structure is also dose dependent: appropriate levels may improve dough viscoelasticity and gluten network connectivity, whereas higher levels may reduce specific volume because of water redistribution and disruption of network formation (24). Therefore, the significant decreases in specific volume and loaf height in the 2 and 3% CRP powder groups may have resulted from the combined effects of gluten dilution, competition for water by fiber/pectin, and disruption of gluten network continuity. Structurally, weakening of the continuous gluten network would reduce the extensibility and stability of the films surrounding gas cells during proofing and baking, thereby limiting gas-cell expansion and increasing the likelihood of cell coalescence or collapse. This would result in a lower gas-holding capacity and, ultimately, a smaller loaf volume and denser crumb structure. However, the specific microstructural changes require further verification through dough rheology, microscopy, or protein-conformation analysis. Other studies have shown that limonene may affect yeast fermentation in limonene-containing media or orange peel hydrolysate systems (25), although whether a similar effect occurs in the solid dough system used here remains to be verified.

**Table 2:** Effects of *Citri Reticulatae Pericarpium* powder addition on baking quality attributes of toast bread.

CRP powder addition	Specific volume/(cm^3^/g)	Loaf height/cm	Loaf weight/g	Baking loss/%
A(0%)	2.98 ± 0.02^a^	10.57 ± 0.11^a^	459.70 ± 4.89^bc^	9.86 ± 1.00^ab^
B(1%)	2.96 ± 0.01^a^	10.53 ± 0.15^a^	453.90 ± 1.46^c^	11.00 ± 0.29^a^
C(2%)	2.74 ± 0.06^b^	10.20 ± 0.20^b^	465.63 ± 3.39^ab^	8.70 ± 0.66^bc^
D(3%)	2.54 ± 0.03^c^	10.17 ± 0.15^b^	466.40 ± 1.50^a^	8.55 ± 0.29^c^

A, B, C, and D represent toast bread samples containing 0, 1.0, 2.0, and 3.0% CRP powder, respectively. Data are expressed as mean ± standard deviation (*n* = 3). Different lowercase letters within the same column indicate significant differences among treatment groups (*p* < 0.05), whereas values sharing at least one lowercase letter are not significantly different (*p* ≥ 0.05).

Unlike specific volume and loaf height, loaf weight and baking loss showed a non-linear dose-dependent pattern. At 2 and 3% CRP addition, baking mass loss tended to decrease relative to the 1% group, particularly at 3%. This pattern may be associated with the water-binding properties of fiber and pectin in CRP powder (8), although water-holding capacity was not directly measured in the present study.

### 3.3 Color analysis

As shown in Table 3, CRP powder addition significantly affected crumb color. With increasing CRP powder addition, L* generally decreased and reached the lowest value in the 3% group, indicating lower crumb lightness at the highest addition level. No significant differences in a* were observed among groups, suggesting little effect on the red-green component. In contrast, b* increased significantly with increasing CRP powder addition, indicating a progressive increase in crumb yellowness. Overall, the effect of CRP powder on toast crumb color was mainly manifested as increased yellowness and, at higher addition levels, darker crumb color, which may be related to the intrinsic color of CRP powder (18).

**Table 3:** Effects of *Citri Reticulatae Pericarpium* powder addition on color parameters of toast bread.

CRP powder addition	L*	a*	b*
A(0%)	67.31 ± 1.53^a^	7.54 ± 0.27^a^	15.68 ± 0.79^d^
B(1%)	65.87 ± 0.76^ab^	7.52 ± 0.16^a^	18.36 ± 0.48^c^
C(2%)	65.81 ± 0.91^ab^	7.36 ± 0.23^a^	19.72 ± 0.31^b^
D(3%)	63.77 ± 1.20^b^	7.60 ± 0.14^a^	23.86 ± 0.17^a^

A-D represent toast bread samples containing 0, 1.0, 2.0, and 3.0% CRP powder, respectively. Data are expressed as mean ± standard deviation (*n* = 3). L* represents lightness, a* the red-green axis, and b* the yellow-blue axis. Different lowercase letters within the same column indicate significant differences among treatment groups (*p* < 0.05), whereas values sharing at least one lowercase letter are not significantly different (*p* ≥ 0.05).

### 3.4 Texture analysis

As shown in Table 4, the CRP powder addition level markedly affected the texture properties of toast bread. Hardness generally increased with increasing addition level, from 7.41 N in the 0% group to 9.37 N in the 3% group, while chewiness increased from 47.18 to 57.80 mJ, indicating a gradual shift from a relatively soft to a more compact texture. In contrast, springiness did not differ significantly among groups, indicating that CRP powder had little effect on the elastic recovery of the toast bread. Cohesiveness decreased slightly, suggesting weaker internal structural integrity at the highest addition level. Combined with the decreases in specific volume and loaf height described above, these results indicate that CRP powder inhibited loaf volume development and produced a more compact crumb structure, which was further reflected by increased hardness and chewiness. From a structural perspective, reduced gas-cell expansion and a less continuous gluten network would increase the relative solid fraction of the crumb and produce smaller or less uniformly developed gas cells with thicker cell walls. Such a compact cellular structure requires greater force for deformation during compression, which is consistent with the observed increase in hardness. Because chewiness is determined jointly by hardness, cohesiveness, and springiness, the increase in hardness was likely the principal contributor to the higher chewiness observed at elevated CRP powder levels. The absence of a significant change in springiness further suggests that CRP powder primarily affected crumb density and resistance to compression rather than completely impairing elastic recovery. Similar behavior has been reported in other fruit-powder-fortified breads, in which higher fruit-powder addition levels were accompanied by decreased specific volume and increased hardness (18). These changes may be associated with reduced continuity of the gluten network after replacement of wheat flour by fruit powder and with water redistribution induced by dietary fiber (23, 24). Overall, although higher CRP powder addition enhanced the characteristic CRP aroma, it also caused the texture of toast bread to shift from soft toward increasingly compact, with the most pronounced effect in the 3% group.

**Table 4:** Effects of *Citri Reticulatae Pericarpium* powder addition on texture properties of toast bread.

CRP powder addition	Hardness/N	Cohesiveness	Springiness/mm	Chewiness/mJ
A(0%)	7.41 ± 0.26^c^	0.67 ± 0.06^a^	10.65 ± 0.12^a^	47.18 ± 4.65^b^
B(1%)	8.05 ± 0.03^b^	0.61 ± 0.05^ab^	10.39 ± 0.23^a^	48.62 ± 3.37^b^
C(2%)	8.37 ± 0.25^b^	0.58 ± 0.05^ab^	10.27 ± 0.86^a^	52.16 ± 2.89^ab^
D(3%)	9.37 ± 0.48^a^	0.56 ± 0.04^b^	10.16 ± 0.41^a^	57.80 ± 2.68^a^

A-D represent toast bread samples containing 0, 1.0, 2.0, and 3.0% CRP powder, respectively. Data are expressed as mean ± standard deviation (*n* = 3). Different lowercase letters within the same column indicate significant differences among treatment groups (*p* < 0.05), whereas values sharing at least one lowercase letter are not significantly different (*p* ≥ 0.05).

### 3.5 Electronic-nose analysis

To facilitate comparison of relative responses among samples for each sensor, the raw electronic-nose data were independently normalized to 0–1 for each sensor, and the mean of replicate samples within each treatment group was used to construct the heat map. Thus, color intensity reflects the relative response of the same sensor among treatment groups rather than the absolute response among different sensors. As shown in Figure 2I, the sensor-response patterns differed markedly among toast bread samples containing different levels of CRP powder. The overall color distribution of the 0% group differed substantially from those of the CRP-supplemented groups, indicating that CRP powder markedly changed the overall odor characteristics of toast bread. The 1% group showed relatively strong responses for LY2/gCT, P30/1, and T40/2, indicating that even a small amount of CRP powder altered the odor composition. The 2% group showed pronounced responses for P40/1, P10/1, T70/2, and P30/2, whereas the 3% group responded strongly on LY2/LG, TA/2, T40/1, and P10/2. Overall, the electronic-nose response did not change monotonically with increasing CRP powder addition; rather, different sensors showed distinct response patterns, reflecting marked changes in the overall odor profile of the samples.

**Figure 2:**
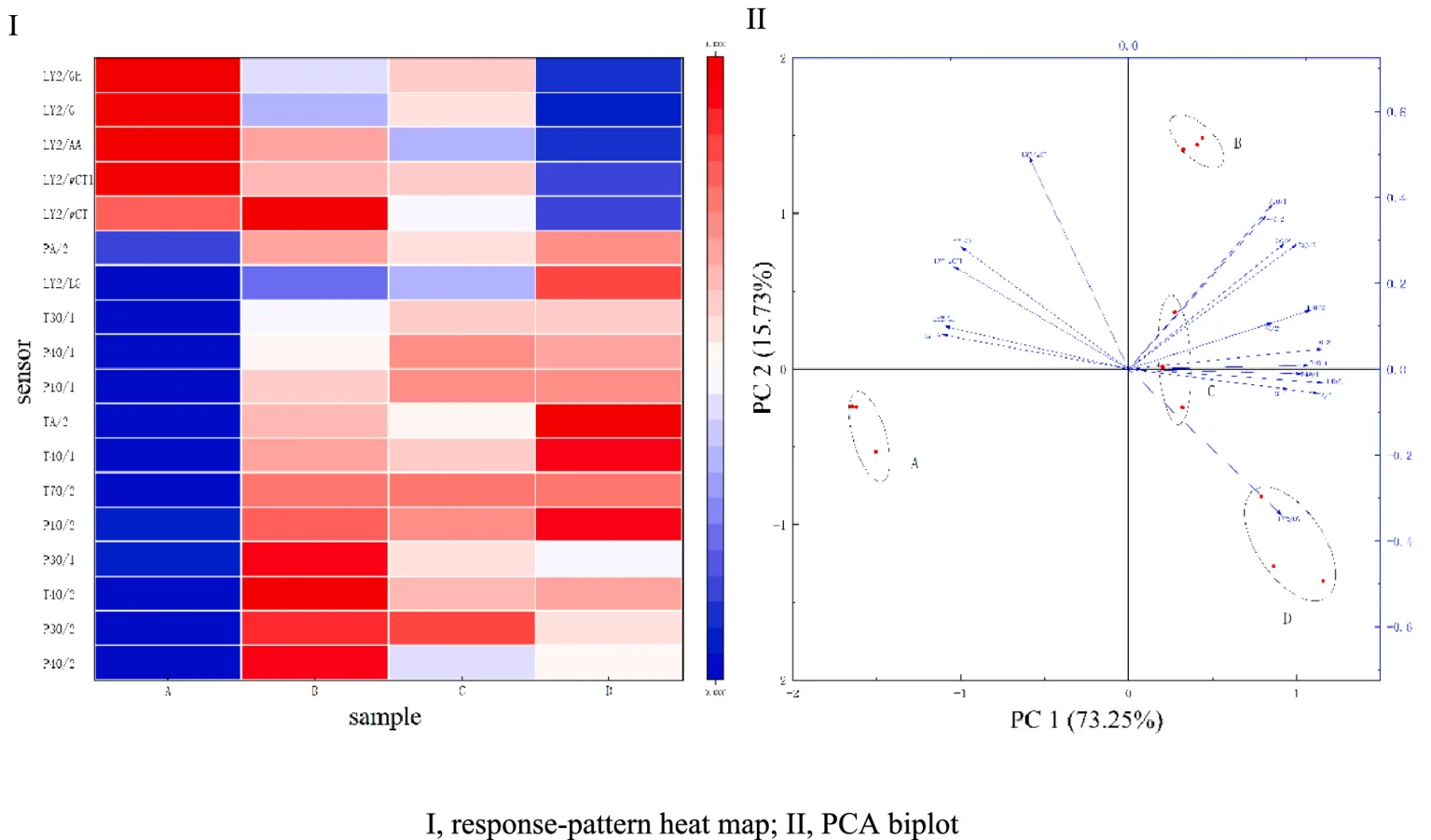
Effects of *Citri Reticulatae Pericarpium* powder addition on electronic-nose response characteristics of toast bread. **(I)** Response-pattern heat map. **(II)** PCA biplot. A, B, C, and D represent toast bread samples containing 0, 1.0, 2.0, and 3.0% CRP powder, respectively. **(I)** The electronic-nose response-pattern heat map. Data from each sensor were independently normalized to 0–1 and are presented as the mean of replicate samples within each treatment group. Color intensity reflects only the relative response of the same sensor among treatment groups and should not be used to compare absolute responses among different sensors. **(II)** The PCA biplot. The variance contributions of PC1 and PC2 were 73.25 and 15.73%, respectively, with a cumulative contribution of 88.98%.

Based on the differences in sensor-response patterns revealed by the heat map, PCA was further applied to reduce the dimensionality of the electronic-nose data and characterize differences in the overall odor profiles of toast bread containing different levels of CRP powder. As shown in Figure 2II, the first two principal components explained 88.98% of the total variance, with PC1 and PC2 accounting for 73.25 and 15.73%, respectively, indicating that these two components captured most of the odor information among the samples. The PCA biplot showed clear separation between the 0% group and all CRP-supplemented groups along PC1, indicating that CRP powder addition was the main factor driving changes in the overall odor profile of toast bread. The 1, 2, and 3% groups were further separated along PC2: the 1% group was mainly distributed on the positive side of PC2, the 3% group on the negative side, and the 2% group between them. This indicates that increasing the CRP powder addition level did not simply intensify the odor profile but induced further compositional changes. Overall, PCA demonstrated that CRP powder markedly altered the overall odor profile of toast bread and that samples with different addition levels showed clear separation trends. The loading distribution indicated that different sensors contributed differently to sample separation. P10/1, P40/1, T40/1, P10/2, TA/2, and T70/2 made relatively large contributions to separation along PC1, whereas LY2/G and LY2/Gh showed relatively high loadings in the opposite direction, suggesting that these sensors contributed to discrimination between the control and CRP-supplemented samples. Meanwhile, LY2/gCT, P30/1, P40/2, LY2/LG, P30/2, T40/2, and LY2/AA contributed notably to separation along PC2, indicating differences among samples with different addition levels across multiple odor-response dimensions. Consistent with the heat-map results, sensors with high loadings also showed pronounced response differences among treatment groups, demonstrating good agreement between PCA separation and electronic-nose sensor-response patterns.

### 3.6 GC–MS analysis

#### 3.6.1 Qualitative and semi-quantitative analysis by GC–MS

GC–MS was used for qualitative and semi-quantitative analysis of volatile compounds in the different toast bread samples, and the results are shown in Table 5. A total of 33 volatile compounds were detected by GC–MS, including terpenes, alcohols, aldehydes, esters, hydrocarbons, ketones, furans, and acids. Overall, alcohols accounted for a relatively high proportion in group A, representing 60.88% of the total semi-quantified volatile content. After CRP powder addition, terpenes increased rapidly and became the major volatile class. Total terpene contents in groups B, C, and D were 3,024.16, 6,745.46, and 8,426.43 μg/kg, accounting for 66.07, 87.04, and 85.12% of the total semi-quantified volatile content in each group, respectively. Meanwhile, the proportions of alcohols and aldehydes generally decreased, whereas ester levels increased markedly in the medium- and high-addition groups, indicating that CRP powder shifted the volatile flavor profile of toast bread from one dominated by fermentation- and baking-related background components toward a composite aroma system dominated by CRP-derived terpene notes.

**Table 5:** Qualitative and semi-quantitative analysis of volatile compounds by GC–MS (Unit: μg/kg).

No.	CAS	Compound	RI	Reference RI	Identification	A	B	C	D	Class
1	18172-67-3	(−)-β-pinene	978	978	MS, RI	–	–	33.72 ± 3.35^b^	246.98 ± 59.29^a^	Terpene
2	99-86-5	α-terpinene	1,017	1,016	MS, RI	–	–	–	6.00 ± 1.05^a^	
3	5989-27-5	D-limonene	1,030	1,030	MS, RI	–	2808.05 ± 407.61^b^	6256.15 ± 1002.62^a^	7572.33 ± 781.78^a^	
4	99-85-4	γ-terpinene	1,058	1,060	MS, RI	–	59.69 ± 6.88^c^	175.02 ± 32.37^b^	272.57 ± 54.66^a^	
5	586-62-9	Terpinolene	1,088	1,092	MS, RI	–	5.21 ± 0.59^c^	10.99 ± 1.17^b^	15.41 ± 0.99^a^	
6	20307-84-0	δ-elemene	1,351	1,324	MS, RI	–	–	–	4.93 ± 0.44^a^	
7	3856-25-5	α-copaene	1,399	1,394	MS, RI	–	9.40 ± 0.89^a^	–	5.34 ± 0.62^b^	
8	515-13-9	β-elemene	1,411	1,392	MS, RI	–	17.24 ± 2.17^a^	12.22 ± 1.74^b^	15.65 ± 2.52^ab^	
9	535-77-3	p-cymene	1,024	1,020	MS, RI	–	124.57 ± 11.29^b^	257.36 ± 59.78^a^	283.85 ± 52.94^a^	
10	10219-75-7	Eremophilene	1,446	1,486	MS, RI	–	–	–	3.37 ± 0.35^a^	
11	71-41-0	1-pentanol	–	–	MS	980.60 ± 136.47^a^	–	–	–	
12	42072-39-9	(*S*)-(+)-3-methyl-1-pentanol	–	–	MS	11.12 ± 1.97^a^	–	–	–	Alcohol
13	111-27-3	1-hexanol	–	–	MS	76.93 ± 12.30^a^	–	–	–	
14	64-17-5	Ethanol	–	–	MS	78.45 ± 12.93^a^	81.64 ± 10.29^a^	126.77 ± 31.79^a^	98.10 ± 10.23^a^	
15	60-12-8	2-phenylethanol	1,115	1,121	MS, RI	36.09 ± 5.51^a^	33.14 ± 5.90^a^	30.48 ± 1.88^a^	29.48 ± 4.09^a^	
16	21661-97-2	*(Z)-7-decenal*	1,106	–	MS	–	–	-	73.23 ± 9.14^a^	
17	124-19-6	Nonanal	1,107	1,089	MS, RI	96.06 ± 14.61^a^	42.76 ± 4.79^b^	–	–	Aldehyde
18	2463-53-8	2-nonenal	1,165	1,159	MS, RI	72.34 ± 16.02^a^	–	–	–	
19	624-24-8	Methyl pentanoate	–	–	MS	–	–	375.01 ± 41.55^a^	191.95 ± 23.52^b^	
20	107-31-3	Methyl formate	–	–	MS	-	54.58 ± 9.06^c^	149.45 ± 5.08^b^	209.19 ± 32.83^a^	Ester
21	106-70-7	Methyl hexanoate	–	–	MS	25.76 ± 1.77^c^	48.26 ± 2.62^a^	34.18 ± 1.00^b^	24.10 ± 2.64^c^	
22	105-21-5	γ-heptalactone	–	–	MS	–	–	–	33.03 ± 5.90^a^	
23	111-11-5	Methyl octanoate	1,125	1,128	MS, RI	12.51 ± 2.24^a^	–	–	10.17 ± 0.54^a^	
24	123-25-1	Diethyl succinate	1,190	1,179	MS, RI	58.89 ± 8.06^a^	–	–	–	
25	106-32-1	Ethyl octanoate	1,196	1,178	MS, RI	53.20 ± 0.92^a^	19.74 ± 2.48^b^	22.17 ± 2.19^b^	20.88 ± 5.37^b^	
26	124-18-5	Decane	996	999	MS, RI	–	131.97 ± 13.41^a^	12.89 ± 2.08^b^	151.27 ± 24.06^a^	
27	1002-43-3	3-methylundecane	1,169	1,171	MS, RI	54.62 ± 3.27^a^	15.79 ± 2.11^b^	10.16 ± 2.79^b^	12.93 ± 1.93^b^	
28	112-40-3	Dodecane	1,199	1,199	MS, RI	54.37 ± 6.24^a^	23.11 ± 7.10^b^	28.25 ± 4.75^b^	20.76 ± 2.79^b^	
29	106-35-4	3-heptanone	–	–	MS	64.52 ± 11.21^b^	93.73 ± 8.55^a^	37.02 ± 9.96^c^	34.67 ± 7.53^c^	Ketone
30	18641-71-9	2,4-dimethyl-3-heptanone	970	970	MS, RI	–	975.98 ± 165.67^a^	148.51 ± 27.52^c^	528.77 ± 33.65^b^	
31	502-56-7	5-nonanone	1,069	1,073	MS, RI	7.32 ± 0.59^b^	32.25 ± 4.24^a^	15.54 ± 2.15^b^	34.11 ± 4.45^a^	
32	3777-69-3	2-pentylfuran	987	989	MS, RI	–	–	14.39 ± 1.85^a^	–	Furan
33	64-19-7	Acetic acid	–	–	MS	260.75 ± 29.33^a^	–	–	–	Acid

A, B, C, and D represent toast bread samples containing 0, 1.0, 2.0, and 3.0% CRP powder, respectively. Volatile compounds were semi-quantified using 2-methyl-3-heptanone as the internal standard. Data are expressed as mean ± standard deviation (*n* = 3) in μg/kg; “–” indicates not detected. MS denotes identification by searching the NIST 2011 mass spectral library, and RI denotes auxiliary identification using retention indices. Compounds without a reference RI or identified solely by MS are regarded as tentatively identified. Different lowercase letters within the same row indicate significant differences among treatment groups (*p* < 0.05), whereas values sharing at least one lowercase letter are not significantly different (*p* ≥ 0.05). Volatile concentrations are semi-quantitative estimates obtained using 2-methyl-3-heptanone as a single internal standard with an assumed relative response factor of 1.0.

Terpenes were the class of volatile compounds most strongly affected by CRP powder addition. No terpenes were detected in group A, whereas terpenes became the predominant volatile class in the CRP-supplemented groups. These results show that CRP powder shifted the volatile composition of toast bread from the fermentation- and baking-related background profile of the control toward a terpene-dominated characteristic aroma system. Previous studies have shown that the volatile fraction of CRP is dominated by terpenes, with D-limonene generally being the major volatile constituent and γ-terpinene and β-pinene also being important components (12–15). These compounds are closely associated with the characteristic citrus, fresh, and aged aroma notes of CRP. Among individual compounds, D-limonene was by far the predominant terpene in all CRP-supplemented groups, with concentrations of 2,808.05, 6,256.15, and 7,572.33 μg/kg in groups B, C, and D, respectively, accounting for 92.85, 92.75, and 89.86% of total terpenes in the corresponding groups. These results indicate that D-limonene was the predominant CRP-derived terpene and was closely associated with the characteristic aroma profile of the CRP-supplemented toast bread. Meanwhile, γ-terpinene increased from 59.69 μg/kg in group B to 272.57 μg/kg in group D, p-cymene increased from 124.57 to 283.85 μg/kg, and (−)-β-pinene was detected only in groups C and D, reaching 246.98 μg/kg in group D. In addition, α-terpinene, δ-elemene, eremophilene, and other terpenes appeared in the higher-addition groups, further broadening the composition of CRP-related volatile compounds (12–15). Consistent with the sensory results, CRP powder significantly enhanced characteristic CRP aroma intensity, while GC–MS showed substantial increases in CRP-derived terpenes, particularly D-limonene, γ-terpinene, and p-cymene, indicating that the enhanced characteristic CRP aroma was closely associated with the enrichment of these terpene volatiles in the baked bread. Group B already exhibited a clear CRP aroma background, whereas groups C and D showed further increases in terpene abundance and compositional richness. D-Limonene increased significantly from 2,808.05 μg/kg in group B to 6,256.15 μg/kg in group C, whereas the difference between groups C and D was not significant (*p* > 0.05), indicating that increasing CRP powder from 2 to 3% no longer produced a statistically significant increase in D-limonene content. Overall, the marked enrichment of CRP-derived terpenes was closely associated with the shift in the volatile and aroma profiles of toast bread from a conventional baked profile toward a citrus–baked composite profile. Although monoterpenes such as D-limonene are highly volatile and susceptible to thermal loss and oxidation, the oven set temperature does not directly represent the thermal history of the bread crumb. During baking, water evaporation limits the temperature rise within the crumb, whereas substantially higher temperatures occur mainly in the drier crust. Therefore, the relatively high terpene levels detected after baking should not be interpreted as evidence of complete thermal retention. Because terpene concentrations were not determined in the CRP powder and dough before baking, their actual retention rates and thermal losses could not be quantified. The detected levels may reflect both the high initial terpene load introduced by CRP powder and matrix-dependent partitioning and release behavior within the bread system; however, the specific mechanism requires further investigation. In addition to thermal loss, the oxidative stability of these monoterpenes should also be considered. D-Limonene and other citrus-derived monoterpenes may undergo oxidation or other transformations during post-baking storage, potentially altering both their abundance and the balance of citrus-like aroma notes. Because the present GC–MS analysis was performed on freshly baked bread after 1 h of cooling, the results represent the immediate post-baking volatile profile and do not establish the storage stability of the CRP-derived aroma. Further time-dependent analysis would be required to determine the persistence of these characteristic volatiles during storage.

Alcohols and aldehydes are important components of bread volatiles. Alcohols in bread can arise from yeast metabolism and lipid degradation, whereas aldehydes can be generated through lipid oxidation, Strecker degradation, and related reactions (1, 2, 5). In this study, total alcohol content in group A was 1,183.19 μg/kg, markedly higher than the 114.78, 157.25, and 127.58 μg/kg observed in groups B, C, and D, respectively. This difference was largely driven by 1-pentanol, which reached 980.60 μg/kg and accounted for 82.88% of total alcohols in group A but was not detected under the present analytical conditions in any CRP-supplemented group. In contrast, ethanol and 2-phenylethanol remained detectable in all groups, with 2-phenylethanol showing relatively small variations. Therefore, the decrease in total alcohol content should not be interpreted as a general suppression of alcohol formation after CRP powder addition. The marked increase in abundant terpenes after CRP addition, together with changes in the bread matrix, may have affected the headspace partitioning, trapping, and thermal desorption behavior of some volatile compounds, thereby influencing their analytical recovery. Because matrix-matched recovery experiments or complementary extraction methods were not performed, the non-detection of 1-pentanol cannot be attributed solely to changes in its formation or release. In contrast, 2-phenylethanol was detected in all groups and varied little, suggesting that it was relatively less affected by CRP powder addition. Total aldehyde content also generally decreased, from 168.40 μg/kg in group A to 42.76 μg/kg in group B, with no aldehydes detected in group C. Although aldehydes increased again to 73.23 μg/kg in group D, the level remained below that of the control. 2-Nonenal was detected only in group A, and nonanal also decreased markedly in the CRP-supplemented groups, indicating attenuation of the original lipid-oxidation-related aldehyde background. The tentative identification of (*Z*)-7-decenal in group D suggests that the detected aldehyde profile was altered at the highest addition level. It should be noted that, although both alcohols and aldehydes decreased overall, their formation pathways differ and should not be explained by a single mechanism. CRP powder may theoretically influence aldehyde formation or release, but the observed aldehyde changes may also be associated with changes in matrix composition caused by wheat flour replacement, altered volatile release behavior, and volatilization losses during baking. Because TBARS, peroxide value, and fatty acid composition were not measured, the present study cannot directly determine whether CRP powder inhibited lipid oxidation in the toast bread system. Overall, the observed changes in detected alcohols and aldehydes, together with the marked enrichment of CRP-derived terpenes, indicate a shift in the detected volatile profile from the original fermentation- and lipid-related background toward CRP-associated volatile characteristics.

Esters are commonly formed through transformation of alcohols and acyl precursors during yeast metabolism and often contribute fruity and sweet notes, making them important components of bread aroma complexity (1, 2). In this study, total ester content changed from 150.36 μg/kg in group A to 122.58, 580.81, and 489.32 μg/kg in groups B, C, and D, respectively. Thus, group B was slightly lower than the control, whereas the medium- and high-addition groups showed marked increases. Methyl formate increased progressively from 54.58 μg/kg in group B to 149.45 μg/kg in group C and 209.19 μg/kg in group D. Methyl pentanoate was not detected in groups A or B but reached 375.01 and 191.95 μg/kg in groups C and D, respectively, and was an important contributor to the increase in total esters in the medium- and high-addition groups. Overall, appropriate CRP powder addition increased some ester compounds associated with fruity and sweet notes and may have contributed to improved aroma harmony and fullness in toast bread.

#### 3.6.2 OAV analysis based on GC–MS

To preliminarily evaluate the potential contributions of major volatile compounds with available odor thresholds to the overall aroma of toast bread, OAV analysis was performed, and the results are shown in Table 6. Compounds with OAV > 1 are generally considered to have potential contributions to overall aroma, whereas compounds with OAV < 1 may have limited individual contributions but can still influence overall aroma through synergistic or modifying effects (22). As shown in Table 6, the composition of potential odor-active compounds differed markedly among samples containing different levels of CRP powder.

**Table 6:** OAV analysis of volatile compounds.

No.	Compound	Threshold*/(μg/kg)	OAV				Odor description
			A	B	C	D	
1	(−)-β-pinene	4,160	–	–	<1	<1	Piney, resinous
2	D-limonene	34	–	82.59	184.00	222.72	Citrus
3	γ-terpinene	1,000	–	<1	<1	<1	Citrus
4	Terpinolene	200	–	<1	<1	<1	Piney
5	α-terpinene	85	–	–	–	<1	Citrus, floral/herbaceous
6	p-cymene	800	–	<1	<1	<1	Fresh citrus, woody
7	Decane	10,000	–	<1	<1	<1	Hydrocarbon-like
8	Dodecane	10,000	<1	<1	<1	<1	Hydrocarbon-like
9	Ethanol	950,000	<1	<1	<1	<1	Alcoholic
10	1-pentanol	150.2	6.53	–	–	–	Oily, fatty, sweet
11	1-hexanol	5.6	13.74	–	–	–	Resinous, floral, green
12	2-phenylethanol	54.23	<1	<1	<1	<1	Rose-like
13	Ethyl octanoate	40	1.33	<1	<1	<1	Pineapple, apple, brandy-like
14	Methyl formate	190,000	–	<1	<1	<1	Aromatic
15	Methyl pentanoate	20	–	–	18.75	9.60	Piney
16	γ-heptalactone	400	–	–	–	<1	Herbaceous
17	Diethyl succinate	790	<1	–	–	–	Fruity
18	Methyl octanoate	200	<1	–	–	<1	Alcoholic, fruity
19	3-heptanone	160	<1	<1	<1	<1	Fruity, fatty
20	5-nonanone	8.2	<1	3.93	1.90	4.16	Fatty, soapy
21	*(Z)-7-decenal*	25	–	–	–	2.93	Fatty
22	2-nonenal	0.1	723.44	–	–	–	Fatty, waxy, hay-like
23	Nonanal	1.1	87.33	38.87	–	–	Strong fatty note; orange- and rose-like notes upon dilution
24	Acetic acid	99,000	<1	–	–	–	Pungent
25	2-pentylfuran	19	–	–	<1	–	Fruity, green

A, B, C, and D represent toast bread samples containing 0, 1.0, 2.0, and 3.0% CRP powder, respectively. “–” indicates not detected, and “<1” indicates an OAV below 1. OAVs were calculated from the semi-quantitative concentrations in Table 5 and should therefore be interpreted as screening indices rather than absolute measures of aroma contribution. *Odor thresholds were obtained from Leo van Gemert, Odor Thresholds: Compilations of Odor Threshold Values [M]. Beijing: Science Press, 2018: 1–659. The odor thresholds were obtained from literature values mainly determined in water or air and are not matrix-specific for toast bread; therefore, the calculated OAVs should be interpreted as screening indices rather than absolute measures of aroma contribution.

In group A, 2-nonenal, nonanal, 1-hexanol, 1-pentanol, and ethyl octanoate all had OAVs greater than 1. Among them, 2-nonenal and nonanal had OAVs of 723.44 and 87.33, respectively, making them the potentially important odor-active compounds in the control. This suggests that the background aroma of ordinary toast bread was mainly influenced by aldehyde-associated fatty, waxy, hay-like, and slightly orange-like notes. The OAVs of 1-hexanol and 1-pentanol were 13.74 and 6.53, respectively, indicating that green, alcoholic, and fatty notes also contributed to the fermentation and baking background aroma of the control. Ethyl octanoate had an OAV of 1.33 and may have modified the fruity aroma dimension. Bread aroma is known to arise from a combination of alcohols and esters formed during fermentation and aldehydes, ketones, and other compounds generated through lipid oxidation, Maillard reactions, and related processes during baking (1, 2). Thus, the potential odor-active compounds in group A were dominated by aldehydes and alcohols, with a smaller contribution from esters, reflecting the superimposition of fatty, fermented, and mild fruity notes in ordinary toast bread.

After CRP powder addition, the composition of potential odor-active compounds shifted markedly. D-Limonene had OAVs of 82.59, 184.00, and 222.72 in groups B, C, and D, respectively, all far greater than 1 and increasing with CRP powder addition. This indicates that D-limonene was identified as a major potential odor-active contributor associated with the citrus-like and characteristic CRP aroma of the toast bread. Previous studies have shown that the volatile fraction of CRP is dominated by terpenes, with D-limonene generally being the major component and closely associated with citrus and fresh aroma characteristics (12, 13). In contrast, although γ-terpinene, terpinolene, α-terpinene, p-cymene, and (−)-β-pinene were detected in the CRP-supplemented groups, all had OAVs below 1, suggesting limited individual contributions and a greater likelihood of acting as background modifiers of the characteristic CRP aroma. Thus, under the conditions of this study, D-limonene was the principal terpene with an OAV > 1, whereas the other detected terpenes all had OAVs below 1.

In addition to D-limonene, nonanal, methyl pentanoate, 5-nonanone, and tentatively identified (Z)-7-decenal were also screened as potential odor-active contributors in the CRP-supplemented groups. In group B, nonanal and 5-nonanone had OAVs of 38.87 and 3.93, respectively, indicating retention of some fatty and ketone-related background aroma at the low addition level. In group C, methyl pentanoate and 5-nonanone had OAVs of 18.75 and 1.90, respectively, suggesting that ester- and ketone-related notes may have modified the aroma complexity of the 2% group. In group D, methyl pentanoate, 5-nonanone, and *(Z)-7-decenal* had OAVs of 9.60, 4.16, and 2.93, respectively, indicating that fatty, ketone-related, and aldehyde-associated notes still contributed to the overall aroma at the highest addition level. Esters and alcohols are generally associated with fermented, fruity, and floral notes in bread, whereas ketones and aldehydes can contribute to fatty, baked, and other aroma dimensions (1, 2). Therefore, aroma differences among addition levels were related not only to the increase in D-limonene OAV but also to changes in these modifying odor-active compounds.

Combined with the sensory results, the 1% group had already developed a noticeable characteristic CRP aroma while retaining some background fatty notes. In the 2% group, the OAV of D-limonene increased substantially, while methyl pentanoate and 5-nonanone also contributed to aroma modification, which may have favored greater aroma fullness and harmony. In the 3% group, the OAV of D-limonene increased further and fatty compounds such as *(Z)-7-decenal* appeared. Although the characteristic CRP aroma was enhanced, these changes may have contributed to lower aroma purity and harmony. Overall, OAV analysis indicated that CRP powder shifted the potential odor-active profile of toast bread from a background aroma system dominated by aldehydes, alcohols, and esters in the control to a characteristic CRP composite aroma system centered on D-limonene and modified by esters, ketones, and other volatiles. Among the CRP-supplemented samples, the 2% group showed a relatively favorable balance between characteristic CRP aroma intensity and overall aroma harmony.

It should be noted that the volatile compounds in this study were semi-quantified using 2-methyl-3-heptanone as a single internal standard, and compound-specific response factors were not available. Differences among volatile compounds in headspace partitioning, trap adsorption and desorption behavior, and MS response may therefore introduce uncertainty into the semi-quantitative concentrations and the OAVs derived from them. Accordingly, the OAVs reported here should be interpreted primarily as screening indices for identifying potential odor-active compounds and comparing their relative changes among treatment groups, rather than as precise quantitative measures of the actual aroma contribution of individual compounds. Further studies using authentic standards and compound-specific calibration curves would be required to more accurately quantify the contributions of major odor-active compounds.

Another limitation concerns the matrix dependence of odor thresholds. The odor thresholds used in this study were mainly obtained from literature values determined in water or air, whereas toast bread is a complex solid matrix containing gelatinized starch, gluten proteins, and lipids. These matrix components can alter the partitioning, retention, and release of volatile compounds. In particular, hydrophobic terpenes such as D-limonene may interact with lipid and protein fractions, thereby reducing their effective release from the bread matrix and altering their apparent sensory thresholds compared with those determined in simpler media. Therefore, the OAVs calculated using literature thresholds should be interpreted as screening indices for potential odor-active compounds rather than as absolute measures of their sensory potency in the bread matrix. Matrix-specific odor thresholds, aroma recombination/omission experiments, or matrix-matched quantitative approaches would be required to more accurately determine the actual aroma contribution of these compounds.

#### 3.6.3 PLS-DA based on OAV data

PLS-DA was performed using the OAV data of replicate samples from each treatment group as the variable matrix. The overall model parameters were R^2^X(cum) = 0.954, R^2^Y(cum) = 0.992, and Q^2^(cum) = 0.979, indicating strong explanatory power for both X and Y variables and high predictive ability. In the score plot (Figure 3I), the first two components explained 63.1 and 17.5% of the X-variable information, respectively, for a cumulative explained variance of 80.6%, adequately representing differences in OAV variables among the treatment groups. Groups A, B, C, and D formed clear clusters, replicate samples within each group were tightly distributed, and all samples fell within the Hotelling’s T^2^ 95% confidence ellipse, indicating good repeatability and no obvious outliers. Along the first component, groups A and B were clearly separated from groups C and D, indicating marked changes in the OAV-variable composition as CRP powder addition increased from low to higher levels. Further separation among treatment groups occurred along the second component, showing that different CRP powder addition levels remained distinguishable. Overall, the PLS-DA model effectively discriminated toast bread samples containing different levels of CRP powder, indicating that CRP powder addition level was an important factor underlying differences in OAV variables.

**Figure 3:**
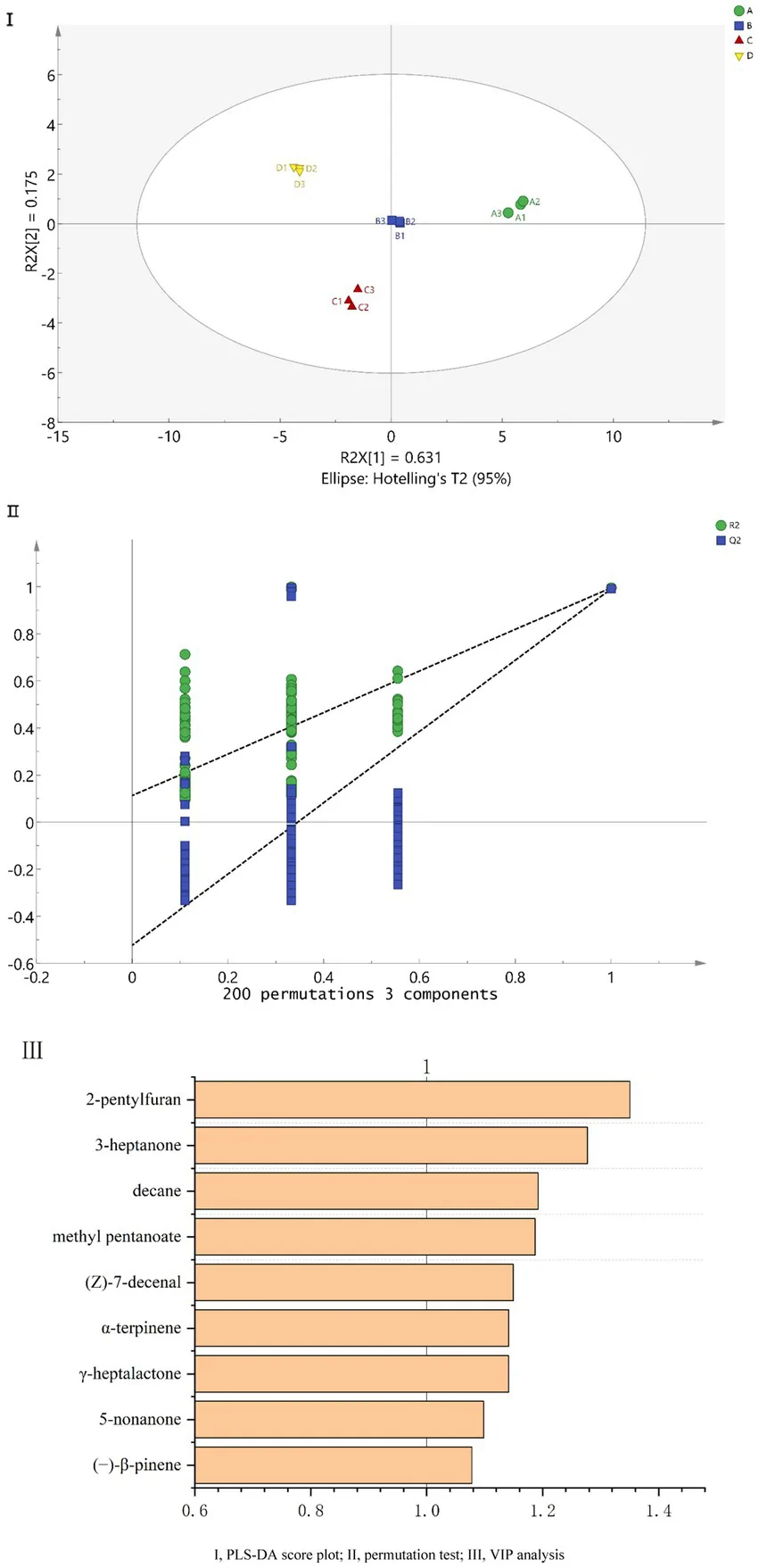
PLS-DA analysis based on OAV data. **(I)** PLS-DA score plot. **(II)** Permutation test. **(III)** VIP analysis. A, B, C, and D represent toast bread samples containing 0, 1.0, 2.0, and 3.0% CRP powder, respectively. **(I)** The PLS-DA score plot established from the OAV matrix of replicate samples in each treatment group; the ellipse represents the Hotelling’s T^2^ 95% confidence interval. **(II)** The 200-permutation test, in which the intercepts of the R^2^ and Q^2^ regression lines on the y-axis were 0.113 and −0.523, respectively. **(III)** The VIP analysis; variables with VIP > 1 were considered to make relatively large contributions to separation among treatment groups.

To further verify the reliability of the PLS-DA model, 200 permutation tests were performed (Figure 3II). The R^2^ and Q^2^ intercepts were 0.113 and −0.523, respectively. In general, when the intercept of the Q^2^ regression line on the y-axis is below 0 and the R^2^ and Q^2^ values of the permuted models are clearly lower than those of the original model, the original model is considered unlikely to result from random classification and the risk of overfitting is relatively low (26, 27). In this study, Q^2^ values of the permuted models were generally markedly lower than that of the original model, and the Q^2^ intercept was negative, indicating good robustness and statistical reliability of the established PLS-DA model and supporting its use for subsequent screening and interpretation of differential OAV variables.

To further screen the major OAV variables contributing to between-group differences, VIP analysis was conducted on the PLS-DA model (Figure 3III). Variables with VIP > 1 are generally considered to contribute substantially to group separation. The results showed that 2-pentylfuran, 3-heptanone, decane, methyl pentanoate, *(Z)-7-decenal*, α-terpinene, γ-heptalactone, 5-nonanone, and (−)-β-pinene had relatively high VIP values and were major differential OAV variables distinguishing toast bread samples with different CRP powder addition levels. Variables with VIP > 1 spanned several chemical classes, including furans, ketones, hydrocarbons, esters, aldehydes, and terpenes, indicating that discrimination among treatment groups resulted from coordinated changes in multiple volatile classes rather than from a single compound.

Notably, although D-limonene had the highest OAV in the preceding analysis and was an important contributor to the characteristic CRP aroma of the CRP-supplemented groups, its VIP value was not among the highest. This suggests that D-limonene was more closely associated with the common characteristic CRP aroma shared by the supplemented groups, whereas variables such as 3-heptanone, decane, methyl pentanoate, *(Z)-7-decenal*, α-terpinene, and γ-heptalactone were more useful for distinguishing differences in OAV profiles among addition levels. Taken together, the score plot, permutation test, and VIP analysis showed that the PLS-DA model based on OAV data effectively discriminated toast bread samples containing different levels of CRP powder, indicating that the effect of CRP powder on toast bread aroma was not simply an enhancement of characteristic CRP aroma but also involved coordinated changes in multiple OAV variables and volatile profiles.

### 3.7 Correlation analysis between electronic-nose responses and volatile compounds

To further establish the relationship between electronic-nose responses and volatile chemical composition, Pearson correlation analysis was conducted between the 18 electronic-nose sensors and the 15 selected volatile compounds, and the results are shown in Figure 4. Two relatively distinct correlation patterns were observed between the volatile compounds and electronic-nose sensors. CRP-related volatiles represented by D-limonene were generally negatively correlated with LY2/G, LY2/Gh, and LY2/gCTl, but showed relatively strong positive correlations with TA/2, T40/1, and P10/2. Specifically, the correlation coefficients of D-limonene with TA/2, T40/1, and P10/2 were 0.949, 0.907, and 0.890, respectively. γ-Terpinene, p-cymene, and methyl formate showed correlation patterns similar to that of D-limonene, indicating that these volatiles, which increased after CRP powder addition, were closely associated with changes in the electronic-nose responses.

**Figure 4:**
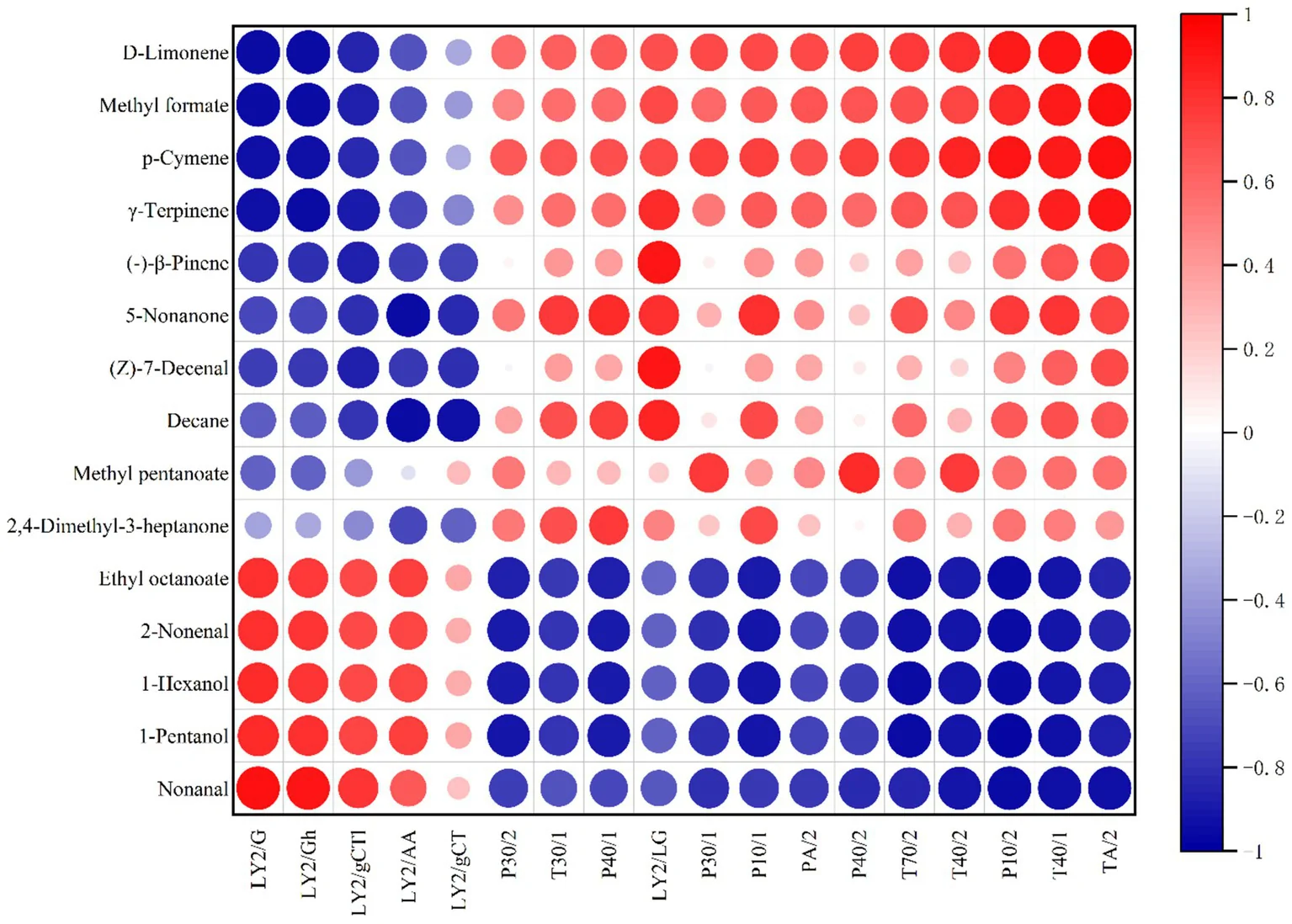
Pearson correlation heatmap between electronic-nose sensor responses and major volatile compounds. The correlation analysis was performed based on 12 one-to-one corresponding independent samples (A1-A3, B1-B3, C1-C3, and D1-D3). Colors represent Pearson correlation coefficients (r); red indicates positive correlation and blue indicates negative correlation, with color intensity reflecting the magnitude of correlation. Volatile compounds included in the analysis comprised potential odor-active compounds with OAV>1 in any treatment group, as well as major differential volatile compounds that were detected in at least two treatment groups, had a semi-quantitative content ≥100 μg/kg in any group, and showed significant inter-group differences (*P <* 0.05).

In contrast, nonanal, 1-pentanol, 1-hexanol, 2-nonenal, and ethyl octanoate, which were relatively abundant in the control group, were strongly positively correlated with LY2/G and LY2/Gh, but negatively correlated with TA/2, T40/1, and P10/2. These results were consistent with the clear separation between the control and CRP-supplemented groups observed in the electronic-nose PCA, indicating that the changes in electronic-nose response patterns after CRP powder addition were closely associated with the shift in volatile composition from background alcohols and aldehydes toward CRP-related volatiles represented by D-limonene, γ-terpinene, and p-cymene. Because the FOX 4000 sensors exhibit cross-sensitivity, these correlations primarily reflect statistical associations between changes in sensor responses and volatile chemical composition and should not be interpreted as evidence that the response of a specific sensor is attributable to a single compound.

## 4 Conclusion

This study systematically investigated the effects of CRP powder addition level on the baking quality and aroma characteristics of toast bread. Increasing CRP powder addition generally decreased specific volume and loaf height, increased hardness and chewiness, decreased crumb L*, and increased b*, indicating that CRP powder inhibited loaf volume development to some extent and darkened product color, while the observed texture changes were consistent with a denser crumb structure. Sensory evaluation showed that CRP powder significantly enhanced the characteristic CRP aroma, with the 2% group performing relatively well in aroma harmony, purity, and fullness. Electronic-nose analysis demonstrated marked differences in the overall odor profiles of samples with different addition levels. GC–MS detected 33 volatile compounds, and CRP powder addition shifted the volatile composition from a profile dominated by fermentation- and baking-related background compounds toward one dominated by terpene-derived characteristic aroma represented by D-limonene. OAV analysis further showed that the potential odor-active profile shifted from fatty- and fermentation-related compounds in the control to a characteristic CRP composite aroma system centered on D-limonene and modified by other volatile compounds. Considering overall quality and aroma characteristics, 2% CRP powder was a relatively suitable addition level, imparting a noticeable characteristic CRP aroma while maintaining relatively good aroma harmony. However, this study did not further verify the underlying mechanisms through dough rheology, microstructural analysis, or calibration curves for key volatile compounds. The mechanisms by which CRP powder affects gluten-network formation and aroma release therefore require further investigation.

## Data Availability

The original contributions presented in the study are included in the article/supplementary material, further inquiries can be directed to the corresponding authors.
